# Sulphur

**Published:** 1887-09

**Authors:** J. T. Kent


### SULPHUR (BRIMSTONE, FLOWERS OF SULPH.).

### A Lecture by Professor J. T. Kent, M. D.

Sometimes a patient will come to your office and say, "Doctor, I want the best medicine you have in the shop, no matter what it costs." Give him Sulph. Nine cases out of ten it will be indicated. Nearly one-half your prescriptions for chronic cases will be Sulph. Yet it is frequently indicated in acute diseases.

From a non-pathological standpoint people seem to be born with certain diatheses which modify any disease which attacks them. In this western country we seem to have one not mentioned in the books—the malarial diathesis. Women suffering from this diathesis while enduring the normal pangs of pregnancy seem to impart to their children this tendency to certain conditions. We also have the scrofulous diathesis, having its origin in part in psora; the hysterical diathesis, also originating in psora, and profoundly reflected upon the entire nervous system, and so on interminably.

Sulph. goes deep into the life, searching for all the weak organs. One never recovers from the effects of a thorough poisoning by Sulph. People who take this remedy every spring, or the same with molasses every night for months, dispose their lives pre-eminently to the chronic effects of Sulph.

Sulph. corresponds to long, lasting chronic diseases, hence its value in homœopathically preventing and curing them. At the close of an acute disease we often have a lingering condition to which Sulph. corresponds—a pneumonia in which resorption has not been fully established, a scarlet fever after which will often be found a diseased condition of ears and eyes. Sulph. will come in and clear up the case. Acute diseases are self-limited, but, because of this predisposition to run into chronic conditions, we have eruptions, catarrhal and kidney complaints, etc Sulph. is deep-acting enough to reach and cure these conditions, which shows its great depth and field of action. Acon. produces rapid inflammatory changes, but Sulph. goes deeper, producing changes in the tissues themselves.

What are these conditions, and how do they look to the would-be prescriber? Let Sulph. be taken for some time, and we have a broken-down mental condition, as from protracted study or overwork. A philosopher, an old inventor, if you will, who has ruined himself mentally and physically in his continual struggle to materialize an idea with no result, becomes poor, tired, careworn, haggard, and dirty in his habits. Here we have a Sulph. patient. Day after day, night after night, week after week, year after year, he has pursued the *ignis-fatuus* of his brain, always just at the point of discovery, and always as surely eluded. Life to him is a failure, which he cannot see. He grows snappish, irritable, and at fifty years is broken down, careworn, stoop-shouldered, lean, and misanthropic. He wishes to be left alone with the wonderful accumulation of wisdom he has gathered in his search for facts concerning that law which *must exist.* In his researches he imagines he has left all his friends behind, and so has none with whom he may communicate or who can sympathize. Hering calls him "a ragged philosopher," and it is a pretty good name for him at this stage of his existence. Content to be left alone, he is dissatisfied with himself and all the world. Deceived as to the relative value of things, old rags are to him as silk, and silk as rags. He knows not of destruction. If religiously inclined, he is philosophizing about religion and has religious melancholy. He philosophizes about every imaginable thing—even of a pin his query is, Who made it? Who made the man who made the pin? Who made God? and so on, ever hunting for first causes. When at the excess of his fantasies he will dream all night of the events of the day, perpetual motion inventors, being unable to recognize that there is a strong opposing law to the one they *suppose* exists. Every shade or degree of mental derangement that may lead to such condition of a patient is covered by Sulph. An old fellow, ragged and dirty, came to me for a prescription. After a dose of Sulph. he came again to the office, this time with a *clean shirt.*

Children, in a measure, begin in this state. They are lean and hungry, look tired, old, and dirty. The dirty little imps one sees running about the streets nearly all need Sulph. Children who go about the streets with dirty noses, who have no need of handkerchiefs, their coat-sleeve answering every purpose, they speedily converting it into a looking-glass, never realizing their nastiness.

The skin reflects a large number of Sulph. symptoms and has all sorts of unhealthy appearances—painful boils, scaly eruptions, pustules, and impetigo. In the scalp are yellowish green patches, covered with yellow crusts, filled with vermin. *All* are *agg.* by water, by washing, by being wet. The Sulph. patient dreads to be washed. Child will cry if washed. *All* complaints made worse by water. Purulent ophthalmia and ulceration of the eyelids *agg.* by bathing, by wet applications. Wetting produces burning in the integument, burning even in the healthy-looking skin. The Puls. baby likes to be washed. The Sulph. baby will yell and scream.

The mucous surfaces of mouth and throat are engorged with blood and are red and angry looking. The lips are red, the throat sometimes studded with varicose veins. The meatus urinarius looks pouting and red. The anus is sore. *All* mucous membranes are *agg.* by washing.

The discharges are acrid and burning; there are none but burning and acrid discharges from Sulph. Burning muco-purulent discharges from eyes, nose, urethra (both male and female), and from the vagina. Liquid fæces (may be constipated) burn violently. Urine burns the meatus. "Excretions burn the parts they pass"—a key-note of Hering. The excretions to most remedies are bland, in Sulph. all are acrid. The mucus of the discharge is either thick or thin, white or milky, but *all* are burning.

We have hot flashes alternating with chilliness in relation to the climacteric period. In this condition it is associated with quite a list of remedies, but notably with Sep. and Lach.

It begins with burning; burning of the soles of the feet, compelling her to put them out of bed at night. Heat is unbearable, and the heat of the bed is very annoying. The next prominent symptom is burning on the top of the head, almost like fire. She feels like carrying something *cold* on the top of the head. These, with the hot flashes, are the most prominent symptoms (and the earliest). Burning may be in the cavities and in the mucous membranes. Burning caused by excretions and secretions. Next to Ars., Sulph. as a general rule produces burning, running through all its symptoms.

A grand and striking feature of Sulph. is the "all-gone, hungry feeling" at eleven A. M.; must have his dinner.

As to the distinctive characteristic of Sulph. in relation to the appetite — violent thirst and no appetite — the key-note reads, "drinks much and eats little."

Another characteristic running through this remedy is the predisposition to hemorrhages of an oozing character; chronic oozing, oozing from the nose in the morning, associated with nausea. Sulph. has cured many cases of predisposition to uterine hemorrhages, preventing their recurrence after having been checked by Bell., Sabin., Ipec.; also when the indicated remedy seems no longer able to prevent the bleeding—when it is no longer able to hold.

Sulph. has emaciation of the entire body, also of single parts. The child looks old and withered; it competes with Puls. in the withering of a part or single limb. It is an old saying that the "diseased limb withers."

Deep-seated rheumatic symptoms of all sorts, with rending, tearing pains. These drawing and tearing pains are all made worse by warmth. Most other pains are made better by warmth. Sulph. is of great value in suppuration of the tissues, which it produces. Before pus is formed it will many times abort suppuration, and will hasten matters after its formation. Avoid Sulph. in suppuration of the *lungs* or in the *cavities*, as it will hasten the suppuration process to the endangering or death of your patient. In the same way it will abort felons, but after suppuration has begun it will hasten the process and relieve the pain.

Most symptoms are *agg.* in the morning, after sleep always finding a bad taste in the mouth. One patient came to me saying, "Doctor, my mouth tastes like a high-hole's nest." I believe there are birds that build their nests in holes made high in the tree. Maybe he had tasted one! He feels ugly, and all mental symptoms are *agg.* after sleep. The tongue is heavily coated. The mouth tastes bad in the morning; he feels stiff and sore and aches all over; his stomach symptoms are *agg.* after eating.

Sulph. produces a state like unto septicemia, attended with quick pulse, fever, sweat, and prostration; hence it is of wonderful value in septic conditions, either traumatic or otherwise.

In big-bellied little children always think of Sulph.

In Sulph. there is a vicious longing for beer, brandy, and something to stimulate and make them feel better.

Another peculiar feature of Sulph. is its power to disperse deposits in the tissues, and shows its profound depth of action. In a neglected or badly treated pneumonia, where there is hepatization, Sulph. goes deeply enough into the life to break down the exudation, clear the lung, and cure the case. It competes with Lyc. and Phos. in that respect. It is to chronic diseases what Acon. is to acute; is thus the chronic of Acon. It is to the veins what Acon. is to the arteries.

In reading over the mental symptoms you will find many to be covered by the sluggish condition of the blood, producing softening of the brain. Therefore we have a weak memory, particularly for names. Sulph. is an important remedy in such conditions.

A peculiar symptom in Sulph. is vertigo on passing over the water in a skiff or over a bridge, and so is found a great remedy in sea-sickness. Compare Cocc.

The head symptoms of Sulph. are only characteristic in this particular—a weekly headache coming on every seven days. You may cover all kinds of headache with Sulph. Headache every two weeks—Ars. It will cure many such cases. "Sick headache, very weakening;" "drawing pains in the limbs, *agg.* by warmth of feathers;" "Severe itching on forehead, also on scalp." This itching is covered by many remedies, and is called "voluptuous itching;" the more you scratch the more you want to scratch. Sulph. has relief after scratching the skin nearly off. Burning pain relieved by scratching; violent itching of the scalp, *agg.* in evening and by warmth of bed. Always *agg.* by warmth, even though he may be cold. All complaints *agg.* by damp, cold weather.

The Sulph. patient has a great amount of dandruff on the head, but it is quite unlike *Calc.* When few symptoms are to be found, a dose of Sulph. followed by Calc.

It has slow formation of bone tissue, like Calc., but its own red string symptoms must be tied to all the others to help you to individualize from any other.

